# Evaluation of the Medically Complex Living Kidney Donor

**DOI:** 10.1155/2012/450471

**Published:** 2012-05-13

**Authors:** Yasar Caliskan, Alaattin Yildiz

**Affiliations:** Division of Nephrology, Department of Internal Medicine, Istanbul School of Medicine, 34390 Istanbul, Turkey

## Abstract

Due to organ shortage and difficulties for availability of cadaveric donors, living donor transplantation is an important choice for having allograft. Live donor surgery is elective and easier to organize prior to starting dialysis thereby permitting preemptive transplantation as compared to cadaveric transplantation. Because of superior results with living kidney transplantation, efforts including the usage of “Medically complex living donors” are made to increase the availability of organs for donation. The term “Complex living donor” is probably preferred for all suboptimal donors where decision-making is a problem due to lack of sound medical data or consensus guidelines. Donors with advanced age, obesity, asymptomatic microhematuria, proteinuria, hypertension, renal stone disease, history of malignancy and with chronic viral infections consist of this complex living donors. This medical complex living donors requires careful evaluation for future renal risk. In this review we would like to present the major issues in the evaluation process of medically complex living kidney donor.

## 1. Introduction

Advances in immunosuppressive therapy, refinement in surgical techniques and in public awareness, altruism, and goodwill have allowed an increase in the number of living donor kidney transplantation; whereby, virtually all biological related, unrelated and medically and psychosocially suitable individuals can be considered as donors [1–6]. Live donor surgery is elective and easier to organise prior to starting dialysis than when the renal donor is a cadaver. In addition, living donor transplants have the advantage of being performed with minimal delay, thereby permitting preemptive transplantation (transplantation prior to dialysis). There is also increasing evidence that patients who undergo preemptive transplantation have improved graft survival compared to those who undergo a period of dialysis before transplantation [7]. Because of superior results with living kidney transplantation, efforts including the usage of “medically complex living donors” are made to increase the availability of organs for donation [8]. The term “complex living donor” was used first by Reese et al. [9] in the International Forum on the Care of the Live Kidney Donor which was held on April 2004 in Amsterdam [7]. The objective of this meeting was to develop international consensus on the standard of care and define the responsibility of the transplant community for the live kidney donor [7–9]. The term “complex living donor” is probably preferred for all suboptimal donors where decision making is a problem due to the lack of sound medical data or consensus guidelines. After this meeting, complex living donors were categorized based on certain risk factors (Table 1). The risk of end-stage renal disease (ESRD) in complex living donors is not clear yet. The evaluation of a potential renal allograft in living donors varies from country to country. In this review we would like to present the major issues in the evaluation process of medically complex living kidney donor.

### 1.1. Informed Consent

First of all, to avoid conflict of interests, the proposed donor should be carefully evaluated by a physician not involved in the care of the proposed recipient. The physician must confirm that the patient's wish to donate is voluntary. Informed consent is a core value in living kidney donation. Prior to donor nephrectomy, the potential donor must be informed of [10] the following.

- The nature of the evaluation process.
- The results and consequences/morbidity of testing, including the possibility that the conditions that may be discovered can impact future healthcare, insurability, and social status of the potential donor.
- The risks of operative donor nephrectomy, as assessed after the complete evaluation. These should include, but not be limited to the risk of death, surgical morbidities, changes in health and renal function, impact upon insurability/employability, and unintended effects upon family and social life.
- The responsibility of the individual and the social system in the management of discovered conditions (e.g., if the donor is discovered to have tuberculosis, the donor should undergo treatment, the community has a responsibility to help the donor secure proper care with referral to an appropriate physician).
- The expected transplant outcomes (favorable and unfavorable) for the recipient and any specific recipient conditions which may impact upon the decision to donate the kidney.
- Disclosure of recipient specific information which must have the assent of the recipient.
- Alternative renal replacement therapies available to the potential recipient.

Additionally, the potential donor should be capable of understanding the information presented in the consent process.

## 2. Clinical Assessment of Medically Complex Living Kidney Donor

### 2.1. Elderly Living Donors

Previously, older kidney donors, such as those over 50 years of age, were not considered as suitable. However, the kidneys from such donors are now commonly used if these individuals are in good physical and mental condition and have adequate kidney function. A survey of kidney transplant centers in the United States in 2007 showed that almost 60 percent of centers had no upper-age limit for kidney donors [11]. However, the influence of donor age on the outcome of living donor kidney transplantation is not clear yet. In a retrospective study by Kumar et al. [12], the long-term outcomes of 112 recipients of kidneys from elderly (>55 years) living related donors were compared with 87 recipients who had younger donors (<45 years). The graft and patient survival rates were similar at one year and five years after transplantation between the two groups [12]. There was also no additional morbidity or deterioration of preoperative blood pressure (BP) and renal function at one year in the elderly donors. Another study from the Mayo Clinic by De La Vega et al. [13] also showed similar results. Overall graft survival, patient survival, and death-censored graft survival at three years did not differ significantly between the recipients of older (>50 years) and younger (<50 years) living donor kidney grafts [13]. However, there were also studies that reported conflicting results [14, 15]. In these studies, significantly poorer graft survival rates were found in kidney transplant recipients from elderly donors [14, 15]. In an analysis of 2.540 living donor kidney transplants at the University of Minnesota, Matas et al. [16] identified donor age greater than 55 years to be a significant risk factor for late graft loss. Advanced age can increase the risk for perioperative complications, but there is no data on long-term outcomes for this specific group in the literature [17].

### 2.2. Obesity

Obesity (BMI > 30 kg/m^2^) in proposed living donors is associated with increased risk for surgical complications as well as future medical problems including diabetes, hypertension, nephrolithiasis, glomerular disease with associated albuminuria or overt proteinuria, and ESRD [18–20]. The relative risk for developing ESRD is threefold for a BMI between 30 and 35 kg/m^2^ and nearly fivefold for a BMI of 35–40 kg/m^2^ [18–20]. The following consensus guidelines regarding obese donors were suggested at the Amsterdam Forum on the Care of the Live Kidney Donor [7].

- Patients with a BMI > 35 kg/m^2^ should be discouraged from donating, especially when other comorbid conditions are present.
- Obese patients should be encouraged to lose weight prior to kidney donation and should be advised not to donate if they have other associated comorbid conditions.
- Obese patients should be informed of both acute and long-term risks, especially when other comorbid conditions are present.
- Healthy lifestyle education should be available to all living donors.

According to our transplantation protocol we also advise lifestyle modifications including diet and exercise to our obese donor candidates.

### 2.3. Living Donors with Asymptomatic Microhematuria

Urine dipstick testing in the absence of fever, trauma, menstruation, or intensive exercise should be repeated twice to confirm the presence of microscopic hematuria. Isolated microscopic hematuria (defined as >3–5 urinary sediment red blood cells (RBCs)/HPF) may not be a contraindication to donation. RBCs with glomerular origin have a dysmorphic appearance observed by phase-contrast microscopy and automated RBC analysis. Patients with persistent microscopic hematuria should not be considered for kidney donation unless urine cytology and a complete urologic work up are performed [7]. If urological malignancy and stone disease are excluded, a kidney biopsy may be indicated to rule out glomerular pathology such as IgA nephropathy. Additionally, Suzuki et al. reported the presence of latent mesangial IgA deposits in approximately 16% of biopsies obtained at the time of transplantation from both living and deceased donors otherwise considered healthy [21].

### 2.4. Living Donors with Proteinuria

Proteinuria greater than 250 mg is a marker of glomerular pathology and renal disease. Proteinuria should be assessed as a standard part of the donor evaluation process. The collection should be repeated and its accuracy checked when the result is abnormal. An overestimate of proteinuria should be suspected if total urine creatinine-to-body-weight ratios are greater than 25 mg/kg (>20 *μ*mol/kg), especially in those with low muscle mass. Various laboratories have different normal values of quantitated urine protein, but a consensus was reached in Amsterdam forum to conclude that a 24-hour urine protein of >300 mg is a contraindication to donation [7]. Forum participants also concluded that microalbuminuria determination may be a more reliable marker of renal disease, but its value as an international standard of evaluation for kidney donors has not been determined. The presence of microalbuminuria should preclude donation.

### 2.5. Hypertensive Living Donors

Hypertension has been considered to be a contraindication in potential renal transplant donors. However, the precise risk to donors who have borderline elevation in BP and those with a family history of hypertension has not been conclusively determined. In general, screening for hypertension in a potential donor includes BP measurement on three separate occasions [7, 22]. According to the guideline by Joint National Committee (JNC 7), BP should preferably be measured by ambulatory blood pressure monitoring (ABPM) [22]. Most transplant centers exclude prospective donors with BP ≥ 140/90 mmHg by ABPM from donation. An echocardiogram may be considered to evaluate for cardiac hypertrophy in cases with borderline high BP or abnormalities suggesting cardiomegaly or left ventricular hypertrophy on chest radiograph or electrocardiogram, respectively. Some patients with easily controlled hypertension who meet other defined criteria (e.g., ≥50 years of age, GFR ≥ 80 mL/min, and urinary albumin excretion < 30 mg/day) may represent a low-risk group for development of kidney disease after donation and may be acceptable as kidney donors [7]. The following consensus guidelines regarding hypertensive donors were adopted at the Amsterdam Forum on the Care of the Live Kidney Donor [7].

- Patients with a BP > 140/90 mmHg by ABPM are generally not acceptable as donors.
- BP should preferably be measured by ABPM, particularly among older donors (≥50 years) and/or those with high office BP readings.
- Some patients with easily controlled hypertension who meet other defined criteria (e.g., >50 years of age, GFR ≥ 80 mL/min, and urinary albumin excretion < 30 mg/day) may represent a low-risk group for development of kidney disease after donation and may be acceptable as kidney donors.
- Donors with hypertension should be regularly followed by a physician.

### 2.6. Living Donors with Diabetes Mellitus

Type 2 diabetes mellitus is an increasingly common cause of ESRD. When the recipient has diabetes mellitus, the risk of related donors developing diabetes later in life is a major concern. All potential living donors should have a fasting plasma glucose estimation to exclude undiagnosed diabetes or glucose intolerance. Most transplantation centers regard established diabetes mellitus as a contraindication to living donation, and many centers exclude individuals deemed as high risk. Although little is known as to whether single-kidney status would accelerate the progression of diabetic nephropathy, there were experimental studies suggesting the increased risk of developing diabetic nephropathy after nephrectomy [23]. A study by Silveiro et al. [24] also suggested that nephrectomy in a patient with type 2 diabetes might increase the progression of disease and microalbuminuria. Another study by Seaquist et al. [25] reported increased risk for the development of diabetic nephropathy in the relatives of patients with diabetic nephropathy (type I). The protocol in our institution is to advise lifestyle modifications including weight control, diet, exercise, and tobacco and excessive alcohol avoidance to proposed donors with impaired fasting glucose, impaired glucose tolerance, and other risk factors. High risk factors for developing type 2 diabetes include those with a familial history, a BMI of ≥30 kg/m^2^, women with the history of gestational diabetes, and excessive alcohol use. All other potential living kidney donors related to recipients with diabetes may also have a preexisting increased risk of developing diabetes and diabetic nephropathy [26]. According to International Amsterdam forum on living donor care, individuals with a history of diabetes or fasting blood glucose of ≥126 mg/dL (7.0 mmol/L) on at least two occasions (or 2-h glucose with OGTT ≥ 200 mg/dL (11.1 mmol/L)) should not donate [7]. However, there was also no comment in this forum about the acceptance of any related donor of a patient with diabetic nephropathy [7].

### 2.7. Living Donors with Renal Stone Disease

A history of urinary tract stones is at least a relative contraindication to donation because urinary system stones tend to recur and may cause obstruction of a solitary kidney. However, renal transplantations performed from donors with kidney stones were reported previously [27–29]. According to Amsterdam forum an asymptomatic potential donor with a history of a single stone may be suitable for kidney donation if [7] he possessed the following characteristics.

- No hypercalciuria, hyperuricemia, or metabolic acidosis.
- No cystinuria or hyperoxaluria.
- No urinary tract infection.
- Multiple stones or nephrocalcinosis are not evident on computed tomography (CT) scan.

Contraindications to donation in individuals with urinary stones are (1) nephrocalcinosis on X-ray or bilateral stone disease; and (2) stone types that have high recurrence rates and are difficult to prevent, such as the following.

- Cystine stones that have a high rate of recurrence and a need for urologic procedures in the donor.
- Struvite stones or infection stones that are difficult to eradicate, and thus it is not feasible to transplant a kidney with them into an immunosuppressed patient.
- Stones associated with inherited or other systemic disorders, such as primary or enteric hyperoxaluria, distal renal tubular acidosis, and sarcoid because of the probability of a high rate of recurrence and the risk of renal insufficiency.
- Stones in the setting of inflammatory bowel disease with an increased risk of stones particularly after bowel resection, also increased risk of renal insufficiency.
- Recurrence while on appropriate treatment (i.e., failed therapy).

Asymptomatic potential donor with current single stone may be suitable if [7]

- the donor meets the criteria shown previously for single stone formers and current stone is ≤1.5 cm in size or potentially removable during transplant. An asymptomatic potential donor with no history of calciluria or colic event is found to have a single stone on evaluation may be suitable for kidney donation if [7];

- no metabolic abnormality or urinary infection exists and if multiple stones or nephrocalcinosis are not evident on CT.

Incidental renal stone detected by magnetic resonance or computerized tomography renal angiography is relatively common (7.4%) in the report from the single center by Kim et al. [30]. Authors reported that kidneys from donors with small (median radius: 2 mm) and nonobstructive stones could be acceptable provided with normal metabolic studies. Another important point is the age of the donor. Younger patients have a longer exposure to risk of recurrence. A stone initially detected in a person older than 50 years is unlikely to recur. In contrast, the risk for stone recurrence is higher in donor candidates aged 25–35 years and must be considered during the evaluation process of donors [7, 29].

### 2.8. Living Donors with History of Malignancy

Kidney donor candidates should be screened for both personal and family history of malignancy. They should undergo standard age- and gender-appropriate screening tests as recommended by national organizations. A prior history of the following malignancies usually excludes live kidney donation [7].

- Melanoma, testicular cancer, renal cell carcinoma, choriocarcinoma, hematological malignancy, bronchial cancer, breast cancer, and monoclonal gammopathy [31–34].

A prior history of malignancy usually excludes live kidney donation but may be acceptable if [7].

- The specific cancer is curable and the potential transmission of the cancer can reasonably be excluded. Examples include colon cancer (Dukes A, >5 years ago), nonmelanoma skin cancer, or carcinoma in situ of the cervix.

An oncology consultation is also advised to donor candidates with a history of malignancy during the donor evaluation process.

### 2.9. Living Donors with Chronic Viral Infections

HIV positive status is a contraindication for donation. Donor with a positive hepatitis C Virus (HCV) serology may be only considered for donation to a HCV positive recipient, if the donor PCR is negative, certain genotypes (genotype 4) are treated and eradicated from the donor, and there is no evidence of chronic hepatitis or cirrhosis on liver biopsy. There is no data on live kidney transplantation from HCV positive donors; however, cadaveric renal transplantations from HCV positive donors to HCV positive recipients were reported [35]. In this report, concerns regarding HCV superinfection if a different HCV genotype of a positive donor is transmitted to a recipient were also stated [35].

Although the detection of hepatitis B surface antigen (HBsAg) in a potential kidney donor generally excludes the individual from live kidney donation [36]. In our unit, living related kidney transplantations from donors with a positive HBsAg to HBV negative recipients were performed with lamivudin prophylaxis when the donor is HBV DNA PCR negative and the recipient's anti-HBS titer is > 100 mIU/mL. Another important point is that even if HBsAg is negative, screening for HBV core total antibody (IgM and IgG) should be performed to exclude low levels of HBsAg and escape mutants of HBV not detectable by the current screening assays for HBsAg [7].

### 2.10. Living Donors with History of Tuberculosis

Tuberculosis is a common infection of renal transplant recipients in developing countries. The peak incidence is after the first year of transplantation and mortality is considerable [37]. Active *Mycobacterium tuberculosis *infection is a contraindication for donation because tuberculosis has been transmitted from live kidney donors to their recipients [38]. Although in some centers a past history of pulmonary tuberculosis is a relative contraindication to donation, many kidney transplantations were performed from donors with a past history of pulmonary tuberculosis in several transplant centers as well as in our unit. Some authors suggested that a positive purified protein derivative (PPD) test due to Bacille Calmette-Guérin vaccination may not be helpful to screen a potential live kidney donor. However, a study from our center reported no tuberculosis in renal transplant recipients who had INH prophylaxis for 1 year after transplantation because their and the donors' PPD skin test reading was greater than 10 mm of induration [37]. In our unit, donation of individuals with a past history of pulmonary tuberculosis is also acceptable. Although there is no clear evidence in the literature for preventing reactivation of tuberculosis and risk of liver toxicity, 1-year INH prophylaxis could be administered to kidney recipients from these donors and recipients with past history of tuberculosis.

Urinary tuberculosis is a contraindication for donation. Additionally, in Amsterdam forum it was suggested that donors previously treated for urinary tuberculosis might have dormant tuberculosis within the kidney and thus remain unsuitable for donation [7].

## 3. Conclusion

Because of superior results with living kidney transplantations, efforts including the use of “medically complex living donors” are made to increase the availability of organs for donation. However, in the evaluation process of these proposed donors we should not forget the following four issues: (1) the risk to the donor must be low, (2) the donor must be fully informed, (3) the decision to donate must be independent and voluntary, and (4) there must be good chance of a successful recipient outcome [39].

## Figures and Tables

**Table 1:** Risk factors associated with complex living donor.

Type of risk factor	Example
Evidence of current renal disease	Hematuria, proteinuria, nephrolithiasis
Direct risk for CKD	Hypertension, obesity
Reduced nephron mass	Age ≥ 65 years
Genetic risk factor	Family history of ESRD in 1st relative
Risk factors for a CKD	Diabetes in first degree relative, impaired fasting glucode
Cardiovascular risk factors	Smoking, hyperlipidemia, hypertension
Other	Black race, sickle trait
Combination of previous factors	Hypertensive black patient

CKD: chronic kidney disease and ESRD: end stage renal disease.
